# A Case-Based Monitoring Approach to Evaluate Safety of COVID-19 Vaccines in a Partially Integrated Health Information System: A Study Protocol

**DOI:** 10.3389/fphar.2022.834940

**Published:** 2022-07-13

**Authors:** Norazida Ab Rahman, Ming Tsuey Lim, Fei Yee Lee, Su Miin Ong, Kalaiarasu M. Peariasamy, Sheamini Sivasampu

**Affiliations:** ^1^ Institute for Clinical Research, National Institutes of Health, Ministry of Health, Selangor, Malaysia; ^2^ Clinical Research Centre, Selayang Hospital, Ministry of Health, Selangor, Malaysia

**Keywords:** COVID-19, vaccine safety, adverse events of special interest (AESI), active surveillance, self-controlled, safety surveillance, real world evidence

## Abstract

In response to Coronavirus disease 2019 (COVID-19) global pandemic, various COVID-19 vaccines were rapidly administered under emergency use authorization. Rare outcomes associated with COVID-19 vaccines might be less likely to be captured in clinical trials, leading to a knowledge gap in real-world vaccine safety. In contrast with high-income countries, many low-to-middle income countries have limited capacity to conduct active surveillance, owing to the absence of large and fully-integrated health information databases. This paper describes the study protocol, which aims to investigate risk of prespecified adverse events of special interests following COVID-19 vaccination in a partially integrated health information system with non-shareable electronic health records. The SAFECOVAC study is a longitudinal, observational retrospective study of active safety surveillance using case-based monitoring approach. This involves linkage of several administrative databases and hospitalization data monitoring to identify adverse events of special interests following administration of COVID-19 vaccines in Malaysia. The source population comprises of all individuals who received at least one dose of COVID-19 vaccine. Self-controlled design and vaccinated case-coverage design will be employed to assess risk of adverse events of special interests and determine the association with vaccine exposure. Data on vaccination records will be obtained from the national COVID-19 vaccination register to identify the vaccination platforms, doses and the timing of vaccinations. The outcome of this study is hospitalization for the adverse events of special interests between March 2021 and June 2022. The outcomes will be obtained through linkage with hospital admission database and national pharmacovigilance database. Findings will provide analysis of real-world data which can inform deliberations by government and public health decision makers relative to the refinement of COVID-19 vaccination recommendations.

## 1 Introduction

In response to the Coronavirus disease 2019 (COVID-19) global pandemic, various COVID-19 vaccines that are either in prelicensure clinical trials or that have been authorized by emergency use authorization were rapidly administered worldwide. Data from clinical trials are inherently limited in providing full safety profile of COVID-19 vaccines, as limited sample size and strict inclusion criteria might limit the generalisability of the safety data, given that trial subjects might differ from actual recipients of the vaccines (Barda et al., 2021; Klein et al., 2021). Furthermore, some adverse reaction events may have longer latency than the trial follow-up period. As such, outcomes which are rare or with delayed onset might be less likely to be detected or captured in clinical trials (Kochhar and Salmon, 2020). Therefore, monitoring of the safety of vaccines during the roll-out of COVID-19 vaccines in real-world population, especially at the post-introduction phase, is particularly important to supplement the findings from clinical trials.

With the roll-out of the COVID-19 vaccination programs worldwide, public concerns over the safety of novel COVID-19 vaccines are anticipated (Li et al., 2021). In order to maintain public health confidence in vaccination, it is important to establish safety surveillance systems to monitor and assess the Adverse Events Following Immunization (AEFIs) and Adverse Events of Special Interest (AESIs) during COVID-19 vaccine introduction. The functions of the vaccine safety surveillance systems include early detection, investigation and analysis of adverse events, as well as appropriate and timely response to these vaccine safety issues (World Health Organization, 2021). In Malaysia, reporting of adverse events to the National Pharmaceutical Regulatory Agency (NPRA) involves spontaneous reporting by healthcare providers or consumers using a standardized form (National Pharmaceutical Regulatory Agency, 2016). This passive surveillance system relies on voluntary submission of reports of illnesses after vaccination but is limited by underreporting, which may potentially underestimate the occurrence of adverse events. Therefore, other approaches to complement existing safety surveillance systems are needed to enhance the identification and monitoring of potential adverse events. Active vaccine safety surveillance for COVID-19 vaccine can be implemented through sentinel surveillance and data linkage to collect information about AEFIs in a defined population by actively looking for cases in a continuous, organized process (Davis et al., 2005; Chandler, 2020).

The type and scope of vaccine safety monitoring activities in each country depend on the availability of the resources related with pharmacovigilance surveillance systems (World Health Organization, 2021). For instance, low-and middle-income countries do not have large healthcare administrative databases or capacity that allows the implementation of active safety surveillance (Kochhar and Salmon, 2020). It is also challenging to implement active surveillance and perform linkage of existing healthcare data in Malaysian settings, given the state of suboptimally integrated healthcare system, and absence of linkage capacity for the existing surveillance system (Duszynski et al., 2021). With the implementation of the Malaysia’s National COVID-19 Immunization Program by the government, uptake of COVID-19 vaccines is collected at individual level, thus leading to the establishment of the first national immunization register in the country. This provides an opportunity to conduct population-level active surveillance for vaccine safety. The ‘Case-Based Clinical Safety Monitoring of Unsolicited Adverse Events Following COVID-19 Vaccination (SAFECOVAC)’ project is initiated for the evaluation of safety aspects of COVID-19 vaccination in Malaysia. Through this project, the risk of pre-specified AESIs in patients exposed to COVID-19 vaccines will be assessed through case-based monitoring that combines hospital-based data extraction and linkage of administrative databases with immunization register to generate the study cohort.

## 2 Study Objectives

The study aims to assess the safety of COVID-19 vaccination on the risk of AESIs within prespecified time windows following immunization among population in Malaysia. This study will also evaluate the risks of AESI between different vaccine platforms and in specific subgroups of interest.

## 3 Methods

### 3.1 Setting

The National COVID-19 Immunization Program (*Program Imunisasi COVID-19 Kebangsaan;* PICK) is a national vaccination campaign implemented by the Malaysian government as an approach in curbing the spread of COVID-19 in Malaysia (Suah et al., 2021). The immunization program began on 24 February 2021 over three phases: 1) Phase 1—healthcare workers and frontliners, 2) Phase 2—senior citizens and high-risk groups, and 3) Phase 3—adults aged 18 years and older {Jawatankuasa Khas Jaminan Akses Bekalan Vaksin COVID-19 (JKJAV) [The Special Committee for Ensuring Access to COVID-19 Vaccine Supply (JKJAV)], 2021}. As of December 2021, vaccines that have been licensed and approved for emergency use in Malaysia are: 1) Comirnaty® (Pfizer-BioNTech), 2) CoronaVac® (Sinovac), 3) ChAdOx1-S (Oxford-AstraZeneca), 4) mRNA-1273 (Moderna), 5) Ad26. COV2-S®[Recombinant] (Janssen), 6) Convidecia^TM^ (CanSinoBio), and 7) COVILO (Sinopharm).

Administration of COVID-19 vaccination are managed via the Malaysia Vaccine Administration System (MyVAS) of the Ministry of Health (MOH), with a mobile application, MySejahtera, which was developed by the government to assist in managing and mitigating the COVID-19 outbreaks in the country (Suah et al., 2021). MySejahtera is the official channel that supports the PICK program through provision of vaccination registration, appointment, and issuance of COVID-19 Immunization digital certificate. It also enables the MOH to monitor users’ health conditions and to facilitate contact tracing for COVID-19 so they can take immediate actions in providing the required treatments.

### 3.2 Study Population and Study Design

The SAFECOVAC study is a longitudinal, observational study using retrospective data collected in administrative registers and health databases. The study population comprised all individuals who received at least one dose of COVID-19 vaccine. This study adapted the research protocol from the vaCcine COVID-19 monitoring readinESS (ACCESS) project to monitor COVID-19 vaccines post-introduction (Kawai and Arana, 2020), as well as Surveillance Program under the Center for Biologics Evaluation and Research Office of Biostatistics and Epidemiology (Lufkin et al., 2021). In this study, self-controlled designs and vaccinated case-coverage design will be employed to assess risk of AESI events and determine association with vaccine exposure.

#### 3.2.1 Self-Controlled Designs

Using self-controlled designs, each subject will serve as his/her own control and implicitly adjusting for fixed confounders (Farrington et al., 1995; Weldeselassie et al., 2011; Baker et al., 2015). Events that occurred during the study period will be categorized into risk period or control period based on the occurrence of events relative to the time of vaccination. Two types of self-controlled designs will be used as specified below.

##### 3.2.1.1 Self-Controlled Case Series (SCCS)

The SCCS method compares incidence of events across different risk periods relative to the exposure with incidence during a baseline or control period. In SCCS, observation period will be anchored based on calendar time during the ongoing COVID-19 vaccination program in Malaysia and inclusion of period before and after vaccination.

##### 3.2.1.2 Self-Controlled Risk Interval (SCRI)

SCRI approach is a subtype of SCCS design and utilizes comparison of incidence of outcome of interest in risk period with that in the control period. Only vaccinated subjects with events occurring during these periods are informative for the analysis. Vaccination date will be used as the index date to define the risk and control intervals. The follow-up period will begin at the date of vaccination and complete at the end of pre-specified duration of the control period.

#### 3.2.2 Vaccinated Case-Coverage

For the vaccinated case-coverage design, the observed odds of vaccination in the exposure window for each case is compared with the expected odds of vaccination for each case during the same time period as the case’s exposure window in an external reference population (Théophile et al., 2011; Baker et al., 2015). The reference population comprises of individuals who are similar to the case with respect to risk factors (e.g., age, sex, comorbidities) and at the same time, the individuals also received the same vaccine during the same time period on the day of the outcome onset.

### 3.3 Study Period

This study is currently plan to cover the period from February 2021, following availability of COVID-19 vaccines in the country, until June 2022.

### 3.4 Exposure Measurement

Exposure is defined as receipt of any COVID-19 vaccine dose. For vaccines administered in multiple doses, each individual dose will be evaluated separately. Individual-level data on vaccination will be obtained from the MyVAS database which has nationwide coverage for COVID-19 vaccination status of populations in Malaysia, including individuals vaccinated abroad. The database provides information on vaccine administered including date of vaccination and vaccine type.

### 3.5 Outcome Measurement

#### 3.5.1 Hospitalizations

The primary outcome is hospitalization for the specified medical events as listed in Table 1. These outcome events were selected based on a list of potential AESIs identified for COVID-19 vaccines from Brighton Collaboration and recommendations from NPRA (Safety Platform for Emergency Vaccines, 2020; Ministry of Health Malaysia, 2021). The list will be reviewed and updated for inclusion or omission of codes, if any, for each round of analysis based on published literatures and latest recommendations. The outcome events will be identified from diagnoses using International Classification of Diseases, Tenth Revision, Clinical Modification (ICD-10-CM) codes.

**TABLE 1:** International Classification of Diseases, Tenth Revision, Clinical Modification (ICD-10CM) codes for pre-specified Adverse Events of Special Interest and recommended risk windows.

AESI category	AESI	ICD-10CM code	Risk windows
Neurological	Guillain Barre syndrome	G61.0	1–42 days (Brighton Collaboration, 2021)
–	Generalised convulsion	G40, G41, R56	–
–	Bell’s Palsy	G51.0	–
Haematological	Thrombocytopenia	D69.3, D69.4, D69.5, D69.6, M31.1	1–42 days (Brighton Collaboration, 2021)
–	Coagulative disorder	D65, D68	–
Cardiovascular events	Microangiopathy	M31.1, I78.9, D65	1–28 days (Smeeth et al., 2004; Qudah et al., 2012)
–	Heart failure	I09.81, I11.0, I13.0, I13.2, I31.4, I50	–
–	Stress induced cardiomyopathy	I51.8	–
–	Coronary artery disease	I20.0, I21, I22, I23-I25	–
–	Myocardial infarction*	I21	–
–	Arrythmia	I44, I44.3, I45, I47, I48, I49, R00.0, R00.1	–
–	Myocarditis	I40.0, I40.8, I40.9, I41.0, I41.1, I41.2, I41.8, I51.4	–
–	Pericarditis	I30	–
Cerebrovascular events	Ischaemic stroke	G45.9, I63	1–28 days (Smeeth et al., 2004; Qudah et al., 2012)
–	Haemorrhagic stroke	I60, I61, I62	–
–	Stroke, unspecified	I64	–
Venous thromboembolism	Pulmonary embolism	I26	1–28 days (Simpson et al., 2021)
–	Lower limb venous thrombosis	I80.1, I80.2, I80.3	–
–	Splanchnic thrombosis	I81, I82.0, I82.1, I82.3	–
–	Other venous thrombosis	I82.8, I82.9, I82.2	–
Others	Anaphylaxis	T78, T80.5, T88.6	**

AESI, adverse event of special interest; ICD-10CM, international classification of diseases, tenth revision, clinical modification.

*Subcategory within coronary artery disease specific for myocardial infarction. **All anaphylaxis events recorded throughout the study period will be included and distribution of time interval from vaccination to occurrence of events will be assessed and reported.

Information on the hospitalizations will be sourced from the Malaysian Health Data Warehouse (MyHDW), a centralized health data repository that collects data from all healthcare facilities across Malaysia including episodes of inpatient care at public and private hospitals. The database provides information on dates of admission and discharge, diagnoses, discharge status, and patients’ demographic. Diagnoses were coded using the ICD-10 coding system; all data within MyHDW are monitored and verified by the Health Informatics Centre, Ministry of Health Malaysia (Health Informatics Centre, 2017). The MyHDW database is updated monthly, but there are potential recording delays from data providers. To account for the data accrual delays, hospital admission data will also be obtained from two other sources: 1) sentinel surveillance sites, and 2) national pharmacovigilance database. These additional measures will allow more rapid identification of eligible cases for early analysis until sufficient data accrue in the central database. Eight public tertiary hospitals across Malaysia were selected as sentinel surveillance sites where hospitalization data will be sourced directly from the medical record office of the respective hospitals. Eligible cases will be identified using ICD-10 coded diagnoses and chart review will be conducted for detailed information of admission episodes. The pharmacovigilance database provides data on AEFI reports collected by NPRA through its spontaneous reporting system. All cases that require hospitalization will be identified for inclusion in the present study and include information on date of events, vaccine administered, and patient outcome.

#### 3.5.2 Mortality

Secondary outcome is all-cause mortality in the vaccinated population. Death will be ascertained using death registrations from the National Registration Department of Malaysia. The death registry covers all deaths in Malaysia including deaths of Malaysian citizens abroad. It provides the date and cause of death based on the death certificate issued.

### 3.6 Data Management and Record Linkage

Table 2 summarizes data used in the study. Data will be collected on monthly basis or at pre-arranged intervals from all data source providers to cover all vaccinations and outcome events that occurred during the study period from 1 February 2021 until 30 June 2022. The hospitalization data will be cross-linked to check for overlapping records between difference data sources and only one record for each case will be retained for analysis.

**TABLE 2:** Data sources of the study.

Type	Description
National COVID-19 vaccination register	Data on vaccination records for Malaysian population will be obtained from the register of COVID-19 vaccination, available via national COVID-19 vaccination register (MyVAS). Data elements include patient demographics and comorbidities, date of vaccination, site of vaccination, vaccine dose, and vaccine batch numbers
Hospital-based medical records	All records of hospitalizations for patients who have received COVID-19 vaccines prior to the admission will be reviewed and relevant cases will be identified for subsequent chart extraction. Variables to be extracted from medical records include patient details, diagnoses, date of admission, date of discharge, and discharge status. Risk factors potentially associated with the event and criteria for vaccination eligibility will also be collected for assessment of potential confounders
National hospital discharge database	Malaysian Health Data Warehouse is a central repository or database of hospitalizations at all MOH hospitals and several private hospitals in Malaysia. This administrative database is managed by the Health Informatics Centre, MOH. Information available from this database are patient details, inpatient admission and discharge dates, and diagnoses. Discharge diagnoses are coded according to the International Classification of Diseases (ICD-10CM). The database is updated periodically based on quarterly data submission from hospitals
National pharmacovigilance database	Data on AEFIs collated by the NPRA based on notifications and reports received from healthcare providers via several platforms including NPRA web portal, the Pharmacy Hospital Information System, adverse drug reaction reports, and manual forms. NPRA database also include reports from public (consumer) on AEFIs received via online and offline reporting form. Information available from this database include patient characteristics, vaccine administered, and details of the AEFIs. Data that have been screened and processed by NPRA will be extracted for analysis to ensure consistency of AEFIs classification
National death register	Data from the National Registration Department on all deaths registered in Malaysia. Information available from this database include patient characteristics, date and cause of death
National COVID-19 cases register (e-COVID/COVID-19 line listing)	This register contains data of all COVID-19 confirmed cases in the country and include details on patient demographics, date of positive test, symptoms at diagnosis, geographical location, and presence of comorbidities. The database is consolidated by cases detected from different sources, ranging from entry point screening, targeted screening, pre-admission screening, passive detection in healthcare institutions, to screening among the brought-in dead (Suah et al., 2021)

AEFI, adverse event following immunization; COVID-19, coronavirus disease 2019; ICD-10CM, International Classification of Diseases, Tenth Revision, Clinical Modification; MOH, Ministry of Health; MyVAS, Malaysia Vaccine Administration System; NPRA, National Pharmaceutical Regulatory Agency.

Data collection at the sentinel sites will utilise electronic case report form from Research Electronic Data Capture (REDCap) 10.0.31 (Harris et al., 2009) hosted at Institute for Clinical Research, MOH Malaysia, with the physical server located in a secured location within the compound. The REDCap platform is managed by a system administrator and information technology support personnel for hardware support from Institute for Clinical Research itself.

**FIGURE 1:**
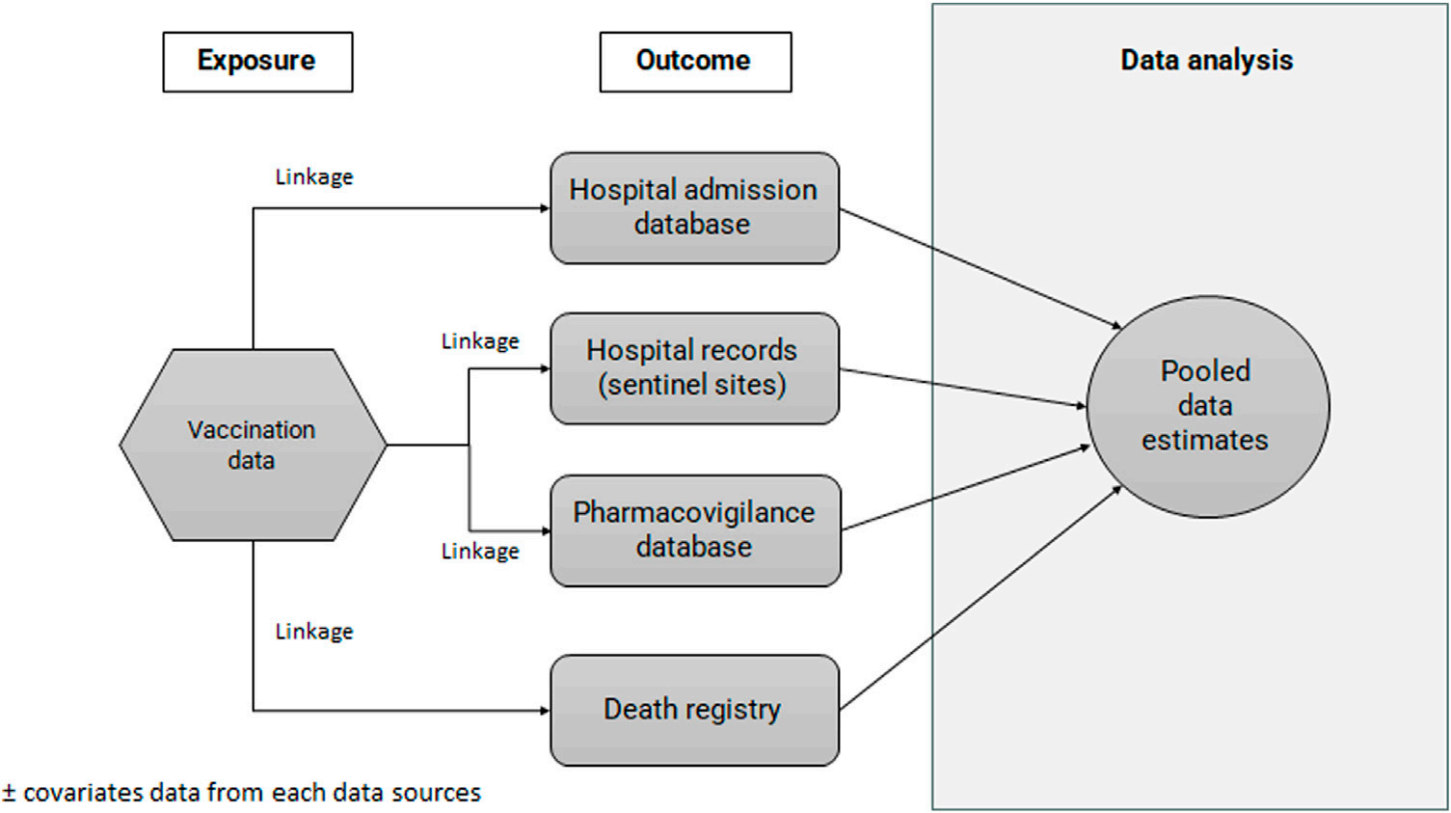
Linkage of data sources for establishment of study cohort.

Each dataset from different repositories will be pooled and linked deterministically with the use of a unique, common patient identifier (Figure 1). This is possible in Malaysia as each person has unique personal identification numbers*.* If shared identifiers are not available, an alternative approach is to use patient-matching algorithms to determine whether two sets of information belong to separate patients or the same patient. However, the matching algorithms requires the availability of some basic data elements such as patient name-first and last, birth date, gender, address and hospital. In situation where data custodians do not allow transfer of individual-level data to the study coordinating centre, the data linkage and analysis will be performed at their sites. Data extraction and linkage will be performed by the research team or data custodians.

### 3.7 Sample Size

The estimated number of cases (i.e., vaccinated cases with an event in the risk or control interval) needed for a self-controlled design to have 80% power under the range of assumed effect sizes and the proportion of the observation period in the risk interval are shown in Table 3 (Musonda et al., 2006). The target sample size to identify a clinically relevant rate ratio of 1.5 for this study was set to 250 events to achieve 80% power. All eligible cases will be included and post-hoc power calculation will be determined based on total cases identified during this study.

**TABLE 3:** Sample size estimation.

Proportion of the observation period in the risk interval (%)	Relative incidence	Sample size (number of cases)
25	0.5	100
25	0.7	350
25	1.5	230
25	2	74
33	0.5	82
33	0.7	292
33	1.5	203
33	2	68
50	0.5	68
50	0.7	249
50	1.5	194
50	2	69

### 3.8 Construction of the Study Cohort

A cohort of patients hospitalized and vaccinated during the study period will be constructed. For patients with multiple hospitalizations for the same diagnosis, only the first hospitalization during the study period will be included in analysis. The cohort will be linked with records of hospital admissions from the previous years to exclude patients who had previous admissions for the same diagnosis within the last 2 consecutive years. COVID-19 infection status will be ascertained by linking the cohort with the database of confirmed cases from the national COVID-19 surveillance database. Patients with a positive COVID-19 test during or within 30 days prior to the admission for the outcome events will be excluded from analysis. Similar method will be applied for mortality outcome. All outcome events that occurred during the study period and fulfilled the criteria will be identified and categorized according to defined time intervals.

### 3.9 Statistical Analysis

Baseline characteristics of the study population will be summarized using descriptive statistics. Categorical data will be reported as counts and percentage while continuous data will be reported as means with standard deviation or medians with range.

#### 3.9.1 Measures of Association

The risk and control period for each outcome event were determined based on review of literature and consultation with subject matter experts (Table 1) (Smeeth et al., 2004; Qudah et al., 2012; Brighton Collaboration, 2021; Lufkin et al., 2021; Simpson et al., 2021). The interval will also take into account vaccine dose interval, depending on vaccine type and dosing schedule.

The self-controlled case-series models will be fitted using a conditional Poisson regression model with logarithm of time in each interval as the offset (Kawai and Arana, 2020). Rates will be calculated based on the number of events during the interval and the amount of time the patient contributes to the interval. Incidence rate ratios and the corresponding 95% confidence interval will be computed for each outcome event.

For vaccination case-coverage method, association between vaccination and outcomes of interest will be calculated as the odds ratio for vaccination in the cases compared with the matched population. This will be computed using logistic regression with the outcome indicating whether vaccination occurred in the case’s exposure or comparison window (Kawai and Arana, 2020). The logarithm of the expected odds of vaccination within the exposure window will be entered into the model as an offset term. The expected probability of vaccination inside the exposure window for each case will be obtained from the reference population.

Stratified models will be used to estimate the risk for subgroups, including but not limited to age and sex stratification. Additional analyses will be undertaken by varying the risk periods and using adjacent risk periods to assess for any clustering of events during a specific period.

### 3.10 Ethics and Dissemination

Study data will be handled and stored responsibly and in accordance with applicable data protection and privacy laws with password protected folders and data files. Data protection and privacy regulations will be observed in collecting, forwarding, processing, and storing of data. No patient-level data will be shared outside of the Institute for Clinical Research, Malaysia or investigation team and the data will not be stored in files that do not meet the requirements of integrity and security. Deidentification of dataset will be performed for statistical analysis. Confidentiality and security protocols will be in place to restrict access to information as appropriate. Study data will be retained for at least five years after completion of the research, archived in a secure server, and destroyed after the specified storage period. Study results will be published following guidelines, including those for authorship, established by the International Committee of Medical Journal Editors (ICMJE, 2021). When reporting results of this study, the appropriate Strengthening the Reporting of Observational Studies in Epidemiology (STROBE) checklist will be followed (von Elm et al., 2008). Study data will not be returned or informed to respective patients. The study results will be reported only in aggregate form and it will not be possible to identify any individual patients from the data that will be presented. No personal information will be disclosed and individual patients will not be identified when study findings are published. The study outcomes will be disseminated through peer-reviewed publications with compliance to the International Committee of Medical Journal Editors guidelines for authorship.

## 4 Discussion

To the best of our knowledge, this study will be among the few from low- and middle-income countries and Asian population on COVID-19 vaccine safety evaluation in real-world environment. Findings from this study will be useful for monitoring development of anticipated AESIs potentially related to COVID-19 vaccination to complement or reaffirm results provided by clinical trials. Furthermore, the findings will provide analysis of real-world data which can inform deliberations by government and public health decision makers relative to the refinement of COVID-19 vaccination recommendations. As the number of vaccinated people around the world increases, growing data from clinical studies including real-world surveillance are important for long-term monitoring of vaccine’s safety and effective profiles in the population (Petousis-Harris, 2020). These data will further form the basis on whether the vaccine is eligible for full approval since all COVID-19 vaccines approved for use in Malaysia are currently available under emergency use authorization by the NPRA with conditional approval (National Pharmaceutical Regulatory Agency, 2020).

For this study, the case-based monitoring approach addresses the potential limitation of underreporting in the existing passive surveillance system to detect AESIs in the population. It consists of active surveillance to find cases rather than relying on voluntary reporting for vaccine safety assessment. Combination of passive and active safety surveillance systems is recommended for monitoring vaccine safety after licensure, but the latter is often time- and resource-consuming (World Health Organization, 2021). However, the increasing availability of automated database and electronic health records led to the approach of using large-linked database for population-based or hospital-based active surveillance for identification and validation of vaccine and drug safety issues based on secondary use of existing data (Scheifele et al., 2002; Huang et al., 2014). The feasibility of conducting active surveillance to quantify adverse events is highly dependent on availability and completeness of data and it is largely applicable to high-income countries where advanced systems and established data sources are readily available. In Malaysia, the integration of the COVID-19 vaccination data into the MyVAS to track vaccine administration enables vaccination status to be linked with other secondary data sources (Suah et al., 2021). This further allows record linkage to be performed across the heterogenous data sources to form a cohort study in a nationwide setting. As such, this study highlights the feasibility of conducting hospital-based active surveillance for vaccine or drug safety evaluations and demonstrate the value of routinely collected healthcare datasets for detecting adverse events. This approach can be considered for enhancement of post approval vaccine and drug safety surveillance systems in the country.

There are several plausible limitations in this study that we acknowledge. First, there is potential misclassification of study variables which is unavoidable in studies utilising secondary data resources. Next, the statistical power to analyse rare outcomes might be limited, given the potential variation in follow-up time periods. Data coverage in the MyHDW hospital admission database for private hospitals is lower compared to public hospitals; however, admission to public hospitals account for nearly 70% of total hospital admissions in the country (Ministry of Health Malaysia, 2019). The use of routinely collected data and administrative database for surveillance are known to be subjected to availability of timely and complete data. For this study, the time-lag associated with obtaining complete data applies to both hospital admission database and death registry. One approach that will be used is to delay the analysis for a period of time until sufficient data becomes available. As such, it limits our ability to conduct a near real-time AESI surveillance. Furthermore, other possible confounders such as disease severity, socioeconomic statuses might not be captured within the existing database and thus, impact of unmeasured confounders could not be evaluated.

In summary, findings from this study will provide insights on the AESIs occurred following COVID-19 vaccination in a real-world setting and contributes to the global data on vaccine safety for comparison. The study protocol provides a framework of a coordinated approach in performing vaccine safety assessment via linkage of several secondary data sources, despite the challenges pertaining to the integration of heterogenous data sources.

## Abbreviations

AEFI, adverse event following immunization; AESI, adverse event of special interest; COVID19, coronavirus disease 2019; ICD-10CM, International Classification of Diseases, Tenth Revision, Clinical Modification; MOH, Ministry of Health; MyVAS, Malaysia Vaccine Administration System; NPRA, National Pharmaceutical Regulatory Agency; PICK, Program Imunisasi COVID-19 Kebangsaan (National COVID-19 Immunization Program); REDCap, Research Electronic Data Capture; SCCS, self-controlled case series; SCRI, self-controlled risk interval; STROBE, strengthening the reporting of observational studies in epidemiology.
